# Vagrant birds as a dispersal vector in transoceanic range expansion of vascular plants

**DOI:** 10.1038/s41598-019-41081-9

**Published:** 2019-03-15

**Authors:** Jesse M. Kalwij, Diego Medan, Jürgen Kellermann, Michelle Greve, Steven L. Chown

**Affiliations:** 1 Centre for Ecological Genomics and Wildlife Conservation, Department of Zoology, University of Johannesburg, Auckland Park, 2006 South Africa; 2 Present Address: Institute of Geography and Geoecology, Karlsruhe Institute of Technology, Reinhard-Baumeister-Platz 1, 76131 Karlsruhe, Germany; 3 Cátedra de Botánica General, Facultad de Agronomía, Universidad de Buenos Aires, Buenos Aires, Argentina; 4 Consejo Nacional de Investigaciones Científicas y Técnicas (CONICET), Buenos Aires, Argentina; 5 State Herbarium of South Australia, Department for Environment and Water, GPO Box 1047, Adelaide, South Australia 5001 Australia; 6 The University of Adelaide, School of Biological Sciences, Adelaide, South Australia 5005 Australia; 7 Department of Plant and Soil Sciences, University of Pretoria, Private Bag X20, Hatfield, 0028 South Africa; 8 School of Biological Sciences, Monash University, Clayton, Victoria, 3800 Australia

## Abstract

Birds are thought to be important vectors underlying the disjunct distribution patterns of some terrestrial biota. Here, we investigate the role of birds in the colonisation by *Ochetophila trinervis* (Rhamnaceae), a vascular plant from the southern Andes, of sub-Antarctic Marion Island. The location of *O. trinervis* on the island far from human activities, in combination with a reconstruction of island visitors’ travel history, precludes an anthropogenic introduction. Notably, three bird species occurring in the southern Andes inland have been observed as vagrants on Marion Island, with the barn swallow *Hirundo rustica* as the most common one. This vagrant displays long-distance migratory behaviour, eats seeds when insects are in short supply, and has started breeding in South America since the 1980s. Since naturalised *O. trinervis* has never been found outside the southern Andes and its diaspores are incapable of surviving in seawater or dispersing by wind, a natural avian dispersal event from the Andes to Marion Island, a distance of >7500 km, remains the only probable explanation. Although one self-incompatible shrub seems doomed to remain solitary, its mere establishment on a Southern Ocean island demonstrates the potential of vagrancy as a driver of extreme long-distance dispersal of terrestrial biota.

## Introduction

Successful long-distance dispersal events are extremely rare, difficult to observe directly, and thus typically only reconstructed by phylogeographic means^1,2^. Notable exceptions are observations of pumice rafting following natural disasters such as tsunamis or volcanic eruptions^3,4^. While the role of abiotic vectors such as oceanic currents and wind in long-distance dispersal events is increasingly acknowledged^5^, biotic dispersal remains difficult to reconstruct^6^. For example, pelagic birds have repeatedly been suggested as a likely long-distance dispersal vector of propagules across oceans^7,8^, although the behaviour of marine birds does not match with the dispersal syndromes of inland terrestrial species^9,10^. Some exceptions do exist however, such as when seabirds come into contact with shoreline vegetation^11^. The mechanisms that underlie long-distance dispersal events, and especially the role of birds therein, is therefore still a matter of ongoing discussion^7,12,13^.

A suitable system to study the potential mechanisms underlying long-distance dispersal events are the Southern Ocean islands. These sub-Antarctic islands are scattered throughout the Southern Ocean and are amongst the most remote places on Earth. Even though the terrestrial biota of these islands are relatively species-poor, they display a pattern of inter-island similarity that indicates long-distance dispersal in an eastward direction^14,15^. This directional dispersal is likely the consequence of strong westerly winds and the West-wind Drift^7,16–18^. Indeed, wind has been suggested as the dispersal vector of recent arrivals of arthropod species to Marion Island^19^. Since sub-Antarctic climate change is resulting in increasing temperatures, the milder climate is additionally resulting in more suitable conditions for propagules of new species to establish as part of natural range expansion^20,21^. However, because the majority of new arrivals to the Southern Ocean islands is the result of anthropogenic dispersal^22^, observations on natural long-distance dispersal events are masked by those of anthropogenic origin, making it difficult to determine whether a new introduction is human-mediated, or the result of a natural dispersal event.

In spite of the remoteness of the Southern Ocean islands from major landmasses, historic and current anthropogenic activities have resulted in a steady rate of colonisation events of non-native species to the islands by means of an anthropogenic vector^23,24^. While not all colonisation events result in successful establishment, often due to the harsh climatic conditions in this region, a large number of species have shown to do extremely well on the islands, often with detrimental impact on the indigenous biota^22,25,26^. Therefore, in recent years an increasing amount of effort has been invested in preventing anthropogenic dispersal of propagules and early eradication of populations of newly introduced species. For example, on South African National Antarctic Programme expeditions no fresh food is allowed, while all cargo, footwear and hiking gear is cleaned and checked for presence of soil and propagules prior to landing. Also, a number of range-restricted alien species are being actively controlled with herbicides^27^. Despite these prevention efforts, some propagules will inevitably slip through and colonise successfully^22,23^.

When a new species previously unknown to a Southern Ocean island is found close to anthropogenic structures such as huts, hiking trails or scientific stations, this typically indicates that the dispersal vector was anthropogenic^20,25^. However, a non-natural dispersal event cannot be assumed *per se*. For example, since propagules of terrestrial biota are unlikely to survive long-term inundation in saline water, wind is the most likely long-distance dispersal vector for some recently discovered populations of wind-dispersed species such as lichens, bryophytes and ballooning arthropods^19,28^. For non-wind-dispersed biota, pelagic and migratory birds are assumed to be the main vectors in natural long-distance dispersal events^9,12,13^. Recent discoveries of plant species that are known to other islands of the South Indian Ocean Province have thus been assumed to have been transported by birds travelling between the Southern Ocean islands^29,30^.

The recent discovery of an unidentified shrub at a remote location on Marion Island, a Southern Ocean island, however, did not fit any of these explanations. The enigmatic appearance of the species indicated that it had originated from outside the South Indian Ocean Province, while the remote location of this small population is contrary to an anthropogenic dispersal event. Since detailed data are available on human activities on this island and on resident and migratory birds, this discovery provides a unique model system to explore an alternative dispersal route.

In this study, we investigate the role of long-distance dispersal events in the colonisation process of Southern Ocean islands. We do this by reconstructing the dispersal route of a recently discovered unidentified shrub on a Southern Ocean island. We first identify the species using a combination of plant anatomy and phylogenetic techniques to determine the region of origin, and assess its arrival time on Marion Island. We then reconstruct historic visitors’ movements to the island to assess whether the dispersal event was of anthropogenic or natural origin. Since birds constituted a potential dispersal vector of the plant species, we compare bird occurrence data from the plant species’ region of origin with those of its new habitat. We then discuss the role of birds, and in particular that of vagrants, as vectors of long-distance dispersal events.

## Results

### Plant morphological analysis

**Figure 1:**
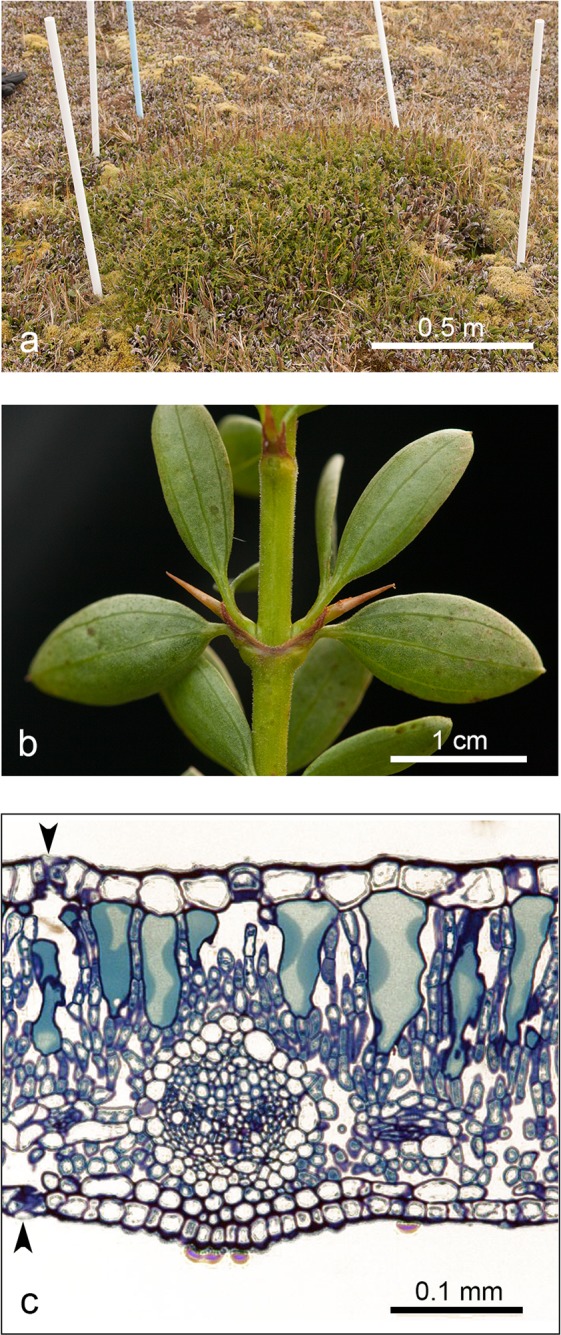
Overview of the plant morphology of *Ochetophila trinervis* on Marion Island: (**a**) some 25 years after establishment, the habitus remains a dwarf shrub; (**b**) detail of a twig showing the 3-nerved abaxial venation pattern; and (**c**) partial transection of a leaf, with arrows indicating the location of epidermis stomata on both leaf sides.

The canopy cover of the unknown shrub had increased from 1.11 m² to 2.09 m² to 2.46 m² between 2004, 2015 and 2017, respectively (Fig. 1a). A linear model fitted to this growth rate indicated — with a 90% confidence interval of 1.6 y (linear model: canopy cover = 0.099 × year – 196.8; F = 59.95, P = 0.082, r²_adj_ = 0.967) — that the shrub had sprouted in late 1992.

The unknown shrub displayed a set of traits typical of the tribe Colletieae (Rhamnaceae): decussate leaves subtending two serial buds each, and an upper bud giving origin to a short spine (Fig. 1b). The connate stipules of opposite leaves, the more or less spatulate leaf shape, the entire leaf margin and the presence of three main veins suggested a close proximity to *Ochetophila trinervis* (Gillies ex Hook. & Arn.) Poepp. ex Endl.^31^, a self-incompatible perennial shrub with wind- and insect-pollinated hermaphroditic flowers^32^. The leaf sections and leaf clearings indicated that the unknown shrub had an anatomical structure similar to *O. trinervis*^33^. The unknown shrub had stomata in the upper and lower leaf epidermis (Fig. 1c). Upper epidermis stomata were also found for the 3-month old *O. trinervis* seedlings, but not for the older specimens.

### Phylogenetic analysis

**Figure 2:**
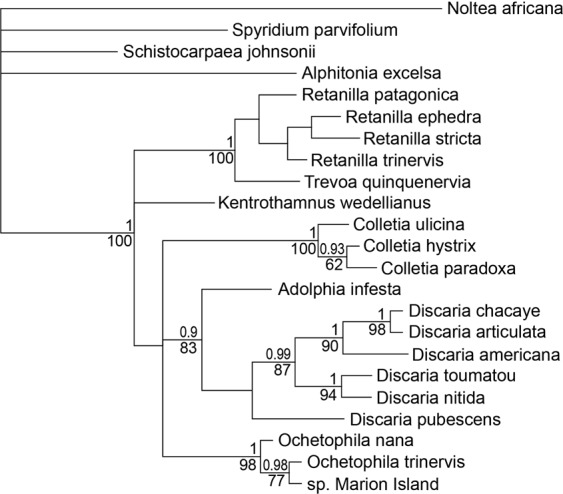
Majority-rule consensus tree (Bayesian analysis) of Colletieae, including sp. Marion Island, based on *trn*L-F sequence data, indicating that the Marion Island species is the same species as the *Ochetophila trinervis* specimen from South America. Numbers above nodes represent posterior probabilities, numbers below are bootstrap values of the ML analysis. Posterior probabilities below 0.80 and bootstrap values below 60% are not indicated.

The final alignment of 24 sequences had a length of 829 bp, of which 638 bp (77%) were identical. The Bayesian and ML analysis of the tribe Colletieae, based on *trn*L-F sequence data, resulted in congruent phylogenies. As such, only the consensus tree of the Bayesian analysis is presented (Fig. 2). The tribe itself received support of 1.00/100% (posterior probability/bootstrap value). The *Trevoa*/*Retanilla* and *Adolphia*/*Discaria* clades, as well as the genera *Colletia* and *Ochetophila* have support of 0.9/80% and above. The unknown shrub is a sister taxon of *O. trinervis* with a support of 0.98/77% (labelled as “sp. Marion Island”). In the final alignment, the sequences of *O. trinervis* and the unknown shrub are identical.

### Biogeographic analysis

**Figure 3:**
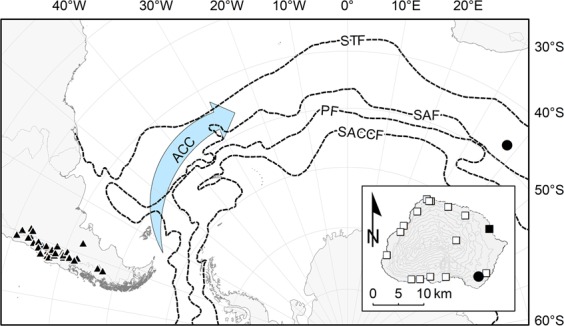
Overview of the known distribution range of *Ochetophila trinervis* in South America (▲) and the location of Marion Island (•) in the sub-Antarctic Indian Ocean (Projection: Lambert azimuthal equal-area for the South Pole). The arrow depicts the Antarctic Circumpolar Current (ACC), whereas the dashed lines represent the major oceanic fronts: Subtropical Front (STF), Subantarctic Front (SAF), Polar Front (PF), and Southern Antarctic Circumpolar Current Front (SACCF). The inset depicts the geographical dimensions of Marion Island (Projection: Transverse Mercator), the location of the permanently manned base (▪), the current and former field huts (▫), and of the only known *O. trinervis* shrub (•).

We collated 47 georeferenced records for *O. trinervis*. All of these observations were located in the lowlands to montane areas of the southern Andes (Fig. 3). No records were found outside this range. Particularly, there were no herbarium records of this species in the online database of the South African National Biodiversity Institute (http://newposa.sanbi.org; date accessed: September 14, 2018). The nearest naturally occurring population was near Lake Pueyrredón (Argentina, 47°26′10″S,71°55′23″W) observed by DM. The geodesic distance between the *O. trinervis* observation on Marion Island and this nearest natural population was ~7530 km. The Euclidean distance from *O. trinervis* to the research station on Marion Island was 9.6 km, and to the nearest field hut 1.6 km (Fig. 3, inset).

### Dispersal analysis

We contacted 35 of 123 persons that were listed as visitors to Marion Island in the period 1986–1991. Of the 18 respondents, all indicated that they had not visited South America prior to their visit to Marion Island. Pre-Marion Island travel history was mostly limited to Namibia and Angola as part of military service.

Chown and Froneman^34^ report 86 bird species for the Prince Edward Islands, which includes all breeding and vagrant species (Supplementary Table S1). Some 56 of these are also listed on the bird checklists of Chile or Argentina. The vast majority of these species, however, have a strictly marine or coastal habitat preference, which does not overlap with the distribution range of *O. trinervis*. Only three species have an inland terrestrial habitat preference and a distribution range that overlaps with *O. trinervis*, namely: cattle egret *Bubulcus ibis*, pectoral sandpiper *Calidris melanotos*, and barn swallow *Hirundo rustica*. Each of these three bird species has a cosmopolitan distribution range, shows migratory behaviour, and has been recorded as a vagrant on Marion Island. All other vagrants on Marion Island with a terrestrial habitat preference possess a breeding range that is strictly limited to the African continent.

## Discussion

We successfully identified a vascular plant new to a South Indian Ocean Province island using a combination of plant morphological comparison and DNA analysis, and estimated its time of arrival to the island. Typically, the discovery of a species new to a sub-Antarctic island is reason for concern, since the vast majority of such discoveries reveal a further spread of non-indigenous species across this biogeographical zone^20,22,35^. Below we discuss the identification process, whether its arrival on the island was the result of an anthropogenic or a natural dispersal event, and how these findings contribute to our understanding of long-distance dispersal events.

Our molecular analysis closely resembled previous phylogenetic classifications of the Colletieae with the genus *Ochetophila* as a distinct and well-supported clade^31,36,37^. Since the DNA sequences and leaf characteristics of the unknown shrub on Marion Island were almost identical to the reference material (Figs 1 and 2), we identified the specimen with high confidence as *Ochetophila trinervis* (Rhamnaceae: Colletieae). This record is remarkable because the natural distribution range of *O. trinervis* is strictly limited to the southern Andes^32,38^. Since only a single individual of *O. trinervis* was found on Marion Island, no genetic diversity analysis could be conducted at the population level to estimate time since arrival. Also, none of the systematic or *ad hoc* biological surveys conducted between 1965 and 2004 revealed a species anything like this one^25^. We are therefore confident that *O. trinervis* is a recent arrival to Marion Island, and that it has gone undetected for some 12 years due to its inconspicuous growth form and remote location on the island. A recent arrival alone, however, does not determine whether an introduction was anthropogenic or natural. While an anthropogenic dispersal event appears likely given that most new arrivals to Southern Ocean islands are exotics^20,39^, and many are transported in propagule form by visitors to the island^23^, the result of natural dispersal or of human-assisted dispersal is ambiguous. For example, *O. trinervis* has never been recorded as a non-indigenous species outside its natural distribution range, whereas most new records of non-native species to the Southern Ocean islands are species that are cosmopolitan or invasive elsewhere^18^. We are therefore cautious to exclude a natural dispersal event *a priori*.

On Southern Ocean islands and Antarctica, non-indigenous species are generally first detected around research stations, field huts or in disturbed habitats^20,40^. Such spatial pattern fits the expectation that newly introduced species first establish in close proximity to their point of introduction^41^, often facilitated by some form of disturbance^25,42^. The *O. trinervis* shrub, however, was found in an undisturbed habitat far from such potential point of introduction (Fig. 3), on an island which, due to its young volcanic substrate, is difficult to traverse. Moreover, non-indigenous species frequently have large distribution ranges, are associated with anthropogenic activities, and have been introduced to multiple areas outside their natural distribution range^22,43^. In its natural distribution range, however, *O. trinervis* — a common shrub or treelet, frequently forming woods of hundreds of individuals — is associated with riverine rather than anthropogenic habitats^44^. Finally, our visitors’ survey indicated that no respondent had visited the southern Andes prior to their Marion Island voyage. Although contemporary visitors to Southern Ocean islands generally have a prior travel-history to other cold-climate areas^18^, during the 1980s travelling between South Africa and South America was costly and cumbersome due to geopolitical circumstances^45,46^. We therefore consider the likelihood that a visitor accidentally (or even deliberately) transported an *O. trinervis* propagule from the southern Andes to Marion Island as highly improbable.

Natural extreme long-distance dispersal events are usually by means of wind dispersal, oceanic currents, or avian dispersal^12,13,47^. The diaspore morphology of *O. trinervis*, a dehiscent explosive fruit capsule containing 1–3 smooth wingless seeds of approx. 2 mm in diameter, as typically observed for species with a ballistic dispersal syndrome^48^, precludes attachment to floating debris, animal fur or human clothing^32,44^. This genus and the family Rhamnaceae have also not been discovered in comprehensive assays of seeds associated with the clothing and equipment of visitors to the sub-Antarctic islands or Antarctic continent^23,49^. Moreover, viable seeds of closely related taxa are likely to sink, while saline water swiftly reduces seed viability, precluding hydrochory as a possible dispersal syndrome^50^. Finally, the location of the shrub on the east-side of the island, corresponds with the area where most avian vagrants are observed^51^. Therefore, transoceanic dispersal via anemochory or hydrochory is highly unlikely; leaving avian dispersal as only parsimonious explanation.

Marine and pelagic birds are often suggested as a likely vector in historic and recent transoceanic dispersal events due to the extreme long distances that these birds can cover^7,8,10^. However, these birds do not have the behavioural traits to acquire an *O. trinervis* diaspore from the southern Andes. Of the three bird species with an inland terrestrial habitat preference and observed both on Marion Island and in Chile/Argentina (Supplementary Table S1), the pectoral sandpiper feeds on arthropods and other invertebrates, while the cattle egret predominantly feeds on insects^52^. Although the barn swallow *Hirundo rustica* is also primarily insectivorous, this species is known to feed on fruits and seeds when insects are in short supply^53^. Indeed, barn swallows are the most common vagrant bird species on Marion Island^51^, and have even been observed as far south as King George Island, Antarctica^54^. Moreover, even though the barn swallow predominantly breeds in the Northern Hemisphere, this species has switched its migratory behaviour by establishing sedentary breeding populations in Argentina as recently as the early 1980s^55^. Finally, although it is unclear whether the barn swallows that have been observed on Marion Island are of the subspecies *Hirundo rustica* subsp. *erythrogaster* — as typically observed in South America — the strong circumpolar winds in easterly direction suggest that vagrancy from South America, facilitated by a storm event, is possible^16,35^. Indeed, weather events and growing populations have been associated with transoceanic vagrancy^56,57^. This unique combination of having recently switched its migratory behaviour and being one of the most common vagrants on Marion Island leaves the barn swallow as the most likely avian dispersal vector between the southern Andes and Marion Island.

For a newly discovered species to be considered as an indigenous species, both the origin of the propagule and its dispersal vector need to be determined^20^. Even though we had no physical evidence of a barn swallow depositing an *O. trinervis* propagule on Marion Island, all other indicators pointed to an avian-mediated natural dispersal exclusively. Indeed, migratory birds have been observed to transport ingested vascular plant seeds across oceans^13^, suggesting that vagrants can play a similar role as a vector of transoceanic colonisation. Interestingly, as a member of the Colletieae, this species can form a symbiosis with nitrogen-fixing *Frankia* bacteria, which can have significant local impact on nutrient-poor soils^44^. Since *O. trinervis* does not readily reproduce vegetatively and, under the current climatic conditions, is unlikely to produce any flowers^32^, the future impact of this single shrub is unlikely to be significant. Until and unless this small population goes extinct, we propose the status of *O. trinervis* as an indigenous species to Marion Island and the Southern Indian Ocean Province.

There is no clear evidence yet linking climatic change with increased natural colonisation rates of Southern Ocean islands^58^. However, climate change is expected to speed up island colonisation across long-distances due to changing migratory behaviour^12,21^. Natural range expansion of a vascular plant from the southern Andes to a Southern Ocean island fits the narrative that vagrancy is an important driver of transoceanic dispersal^56^. In addition, climate barriers are lowering, resulting in the Southern Ocean islands and the Antarctic as increasingly suitable for newcomers^59^. Since propagules of alien species are common and have large ecological niches, vagrant birds are expected to disproportionally disperse alien rather than indigenous species and with increased frequency^60^.

## Methods

### Study area

Marion Island — one of the two Prince Edward Islands (South Africa) — is located ~1750 km south of the African continent in the Southern Ocean (Fig. 3). It is a rugged 293 km^2^-sized oceanic island belonging to the Kerguelen Biogeographic Province^34^. The nearest neighbouring islands to the Prince Edward Islands are Bouvet Island at ~2540 km to the west, and the Crozet Islands at ~1080 km to the east. The Antarctic Circumpolar Current ensures that Marion Island’s climate is cool and wet with an average annual temperature of 6.4 °C and just over 2000 mm of precipitation per annum^34^.

The vegetation of Marion Island is relatively species poor due to a combination of climatic constraints and its remoteness from potential species pools. Still, 23 vascular plant species have been recorded as indigenous to the island with an additional 18 species as introduced by humans^25,27^. The island is characterised by low-growing vegetation such as mire and fernbrake habitats, and fellfield vegetation with cushion-forming *Azorella selago*, bryophytes, and lichens. For a detailed description of the vegetation types on Marion Island see Chown and Froneman^34^.

### Plant morphological analysis

On March 18, 2004, an unknown shrub was discovered by Greg Hofmeyr in fernbrake vegetation on the southeast area of the island (Fig. 3 inset; 37°50′02″E, 46°57′40″S). The shrub’s canopy dimensions were measured and a herbarium specimen was collected. However, the shrub remained unidentified and it has since then been listed as a species with an unclear introduction status^34^.

To obtain a species identification, we collected a small piece of a branch with leaves in May 2015. Part of the sample was preserved in 98% ethanol for morphological analysis. The remainder of the sample was preserved in silica gel for genetic analysis. We also measured the dimensions of the canopy in April of 2015 and 2017 to assess growth expansion since its discovery (Fig. 1a).

A tentative identification, based on colour photos (Fig. 1b), suggested that the unknown shrub belonged to a taxon within the Colletieae (Rhamnaceae). Using standard plant anatomical methods, full-grown, healthy and morphologically representative leaves were either embedded in paraffin and transversally sectioned to study the anatomical structure (Fig. 1c), or cleared to study the distribution of stomata and the nerviation pattern. Results were compared with anatomical data on Colletieae^33^. To estimate the plant-anatomical development stage of the unknown shrub, freshly collected leaf samples from specimens of *Ochetophila trinervis* (syn.: *Chacaya trinervis, Discaria trinervis*) of different ages (3-month and 1-year old saplings, and an adult tree) were collected from the species’ native range (San Carlos de Bariloche, Argentina; legit E. Chaia and S. Alzogaray, October 2016), processed as above, and included in the comparison.

### Phylogenetic analysis

To confirm the species identification, we compared the genetic material of the unknown shrub with sequence data for the *trn*L intron and *trn*L-F spacer region of Colletieae from Aagesen, *et al*.^37^, Burge, *et al*.^61^, Kellermann and Udovicic^62^, and Richardson, *et al*.^36^. All species in the tribe were included with the exception of *Colletia spartioides* Bertero ex Colla and *C. spinosissima* Gmel. Outgroups of related tribes and genera were selected according to the most recent family-wide phylogeny^63^. DNA of the unknown shrub was extracted from silica-dried leaf material using the ISOLATE II Plant DNA Kit (Bioline, London). For *Colletia hystrix*, *Retanilla ephedra* and *Retanilla stricta*, the same DNA extract was used as described in Aagesen, *et al*.^37^. All samples are listed in Supplementary Table S2.

Two PCR amplifications of cpDNA were performed using primers *trn*L c and d, and *trn*L e and f^64^. PCR amplifications were performed in 35 μl reactions using the MyTaq™ HS DNA polymerase kit of Bioline reactions containing: 1.5 μl of each 10 µM primer, 7 μl of 5 × reaction buffer, 0.25 μl of polymerase (1.25 U). The cycling profile was as follows: 2 min at 95 °C, then 37 cycles of 20 s at 95 °C, 20 s at 58 °C, 20 s at 72 °C, with a final extension of 2 min at 72 °C. The quality and approximate size of products were checked by electrophoresis on a 1.5% agarose gel. DNA sequences were generated in both forward and reverse direction from direct sequencing of PCR products through the Beijing Genome Institute (China).

To analyse the molecular data we used Geneious 8.1.9 (created by Biomatters, http://www.geneious.com). After initial automatic alignment, the alignment was refined manually. Sequences for both DNA regions, *trn*L intron and *trn*L-F spacer, were aligned separately. Jagged ends of the alignments were trimmed and both regions were combined for analysis. Polynucleotide regions could not be meaningfully aligned and were excluded from the dataset. Three indel regions were treated as informative and removed after coding. Maximum likelihood (ML) of phylogeny estimation was calculated with the plugin PhyML 2.2.3^65^, using the GTR + I + G model of nucleotide substitution and calculating bootstrap support with 100 replicates, 6 substitution rate categories, and an NNI topology search. The Bayesian analysis was run with the plugin MrBayes 3.2.2^66^ using four Markov Chain Monte Carlo heuristic searches of 1.1 mio generations performed in four independent runs. Subsampling frequency was set to 200, burn-in length to 100,000. The substitution model used was GTR + I + G. Parameter trace files were examined to ensure convergence of separate runs. Posterior probabilities are indicated to illustrate support of nodes.

### Biogeographic analysis

To determine the distance between the nearest naturally occurring population of *O. trinervis* and the location of the unknown shrub on Marion Island, we collated all georeferenced observations of *O. trinervis* and its synonyms into a single geodatabase. For this we obtained records from Tortosa^38^ and GBIF (https://www.gbif.org/), supplemented with personal observations by DM. We specifically searched for the occurrence of specimen outside the expected distribution range. We then measured the geodesic distance between the nearest *O. trinervis* record and the unknown shrub.

### Dispersal analysis

To determine whether *O. trinervis* could have been introduced by means of an anthropogenic vector, we obtained a list of names of visitors to Marion Island from the Antarctic Legacy of South Africa (http://blogs.sun.ac.za/antarcticlegacy/) for the time period 1985–1991. The Antarctic Legacy of South Africa keeps a record of all individuals that have visited the island every year. Since no current contact details for visitors during this time period were available, we contacted as many persons as possible on this list via social media (https://www.facebook.com/groups/marionisland/). We asked when they visited Marion Island and which territories outside southern Africa were visited before their visit to the island. Only if the latter question included South America did we ask which areas in South America had been visited, which areas of Marion Island had been visited, and if gear such as shoes, boots or bags had been used during both visits.

To identify which bird species might be a potential vector for the dispersal of *O. trinervis* to Marion Island, we compared the checklist of bird species from the Prince Edward Islands^34^, with those of the countries in which *O. trinervis* occurs naturally: Chile and Argentina (http://www.museum.lsu.edu/~Remsen/SACCCountryLists.htm). To harmonise species names, we adopted the nomenclature of the IOC World Bird List version 7.1 (http://www.worldbirdnames.org/). We identified bird species as potential dispersers based on the following criteria: a distribution range overlapping with *O. trinervis*, a non-marine habitat preference, and a long-distance migration behaviour. Bird distribution ranges and habitat preferences were obtained from GBIF, while migration and feeding behaviour was assessed from literature.

## Supplementary information

Electronic Supplementary Material
