# Supplementary material for: Vagrant birds as a dispersal vector in transoceanic range expansion of vascular plants
Source: Sci Rep. 2019 Mar 15;9:4655. doi: 10.1038/s41598-019-41081-9 (PMC6420631; doi:10.1038/s41598-019-41081-9)
Supplement: Supplementary file 1 — Electronic Supplementary Material [file 41598_2019_41081_MOESM1_ESM.pdf]

Vagrant birds as a dispersal vector in transoceanic range expansion of vascular plants

Supplementary Information

Jesse M. Kalwij [1,2,*], Diego Medan [3,4], Jürgen Kellermann [5,6], Michelle Greve [7], Steven L. Chown [8]

[*] Email corresponding author: jessek@uj.ac.za

[1] Centre for Ecological Genomics and Wildlife Conservation, Department of Zoology, University of Johannesburg, Auckland Park 2006, South Africa

[2] Institute of Geography and Geoecology, Karlsruhe Institute of Technology, Reinhard-Baumeister-Platz 1, 76131 Karlsruhe, Germany

[3] Cátedra de Botánica General, Facultad de Agronomía, Universidad de Buenos Aires, Buenos Aires, Argentina

[4] Consejo Nacional de Investigaciones Científicas y Técnicas (CONICET), Buenos Aires, Argentina

[5] State Herbarium of South Australia, Department for Environment and Water, GPO Box 1047, Adelaide, South Australia 5001, Australia

[6] The University of Adelaide, School of Biological Sciences, Adelaide, South Australia 5005, Australia

[7] Department of Plant and Soil Sciences, University of Pretoria, Private Bag X20, Hatfield 0028, South Africa

[8] School of Biological Sciences, Monash University, Clayton, Victoria 3800, Australia

Supplementary Table S1. Checklist of birds on the Prince Edward Islands including their occurrence status following Chown and Froneman [1], and whether the respective species is present in Chile (https://www.avesdechile.cl/) or Argentina [2].

Supplementary Table S2. Sources of DNA material used from Aagesen, et al. [3], Burge, et al. [4] Kellermann and Udovicic [5], and Richardson, et al. [6]. Genbank numbers are listed for the *trn*L intron and the *trn*L-F spacer. Where the same number is listed for both DNA regions, this indicates that the full *trn*L-F region is deposited in GenBank. New sequences are indicated in bold and their voucher specimens listed.

References

1 Chown, S. L. & Froneman, P. W. *The Prince Edwards Islands. Land-sea interactions in a changing ecosystem*. 1st edn, i-470 (Sun Press, 2008).

2 Narosky, T. & Yzurieta, D. *Aves de Argentina y Uruguay, Guía de identificación. Edición total*. (Vazquez Mazzini Editores, 2010).

3 Aagesen, L., Medan, D., Kellermann, J. & Hilger, H. H. Phylogeny of the tribe Colletieae (Rhamnaceae) — a sensitivity analysis of the plastid region *trn*L-*trn*F combined with morphology. *Plant Syst. Evol.* **250**, 197-214, doi:10.1007/s00606-004-0204-5 (2005).

4 Burge, D. O. *et al.* Diversification of *Ceanothus* (Rhamnaceae) in the California Floristic Province. *Int. J. Plant Sci.* **172**, 1137-1164, doi:10.1086/662028 (2011).

5 Kellermann, J. & Udovicic, F. Large indels obscure phylogeny in analysis of chloroplast DNA (*trn*L-F) sequence data: Pomaderreae (Rhamnaceae) revisited. *Telopea* **12**, 1-22 (2008).

6 Richardson, J. E., Fay, M. F., Cronk, Q. C. B., Bowman, D. & Chase, M. W. A phylogenetic analysis of Rhamnaceae using *rbcL* and *trnL-F* plastid DNA sequences. *Am. J. Bot.* **87**, 1309-1324, doi:10.2307/2656724 (2000).

Family	Vernacular Name	Scientific Name	Marion Status	Argentina	Chile	Overlap O.trinervis	Habitat	Distribution (GBIF)	GBIF Link
Anatidae	Kerguelen Pintail	Anas eatoni	Vagrant*	0	0	0	Coastal	Kerguelen	https://www.gbif.org/species/2498098
Apodidae	Common Swift	Apus apus	Vagrant*	0	0	0	Inland	Europa, Africa	https://www.gbif.org/species/5228676
Ardeidae	Cattle Egret	Bubulcus ibis	Vagrant*	1	1	1	Inland	Cosmopolitan	https://www.gbif.org/species/2480830
Ardeidae	Yellow-billed Egret	Mesophoyx intermedia brachyrhyncha	Vagrant*	0	0	0	Inland	Australia	https://www.gbif.org/species/2480915
Charadridae	Common Ringed Plover	Charadrius hiaticula	Vagrant*	0	0	0	Coastal	mostly Africa and Europe	https://www.gbif.org/species/9566659
Charadridae	Three-banded Plover	Charadrius tricollaris	Vagrant*	0	0	0	Inland	Africa	https://www.gbif.org/species/2480325
Charadridae	Blacksmith Lapwing	Vanellus armatus	Vagrant*	0	0	0	Inland	Africa	https://www.gbif.org/species/5229136
Chionidae	Lesser Sheathbill	Chionis minor marionensis	4000	0	0	0	Marine	Prince Edward Islands endemi	https://www.gbif.org/species/4352353
Ciconiidae	White Stork	Ciconia ciconia	Vagrant*	0	0	0	Inland	Africa, Europe, Asia	https://www.gbif.org/species/2481912
Columbidae	Cape Turtle Dove	Streptopelia capicola	Vagrant*	0	0	0	Inland	Africa	https://www.gbif.org/species/2495667
Columbidae	Laughing Dove	Streptopelia senegalensis	Vagrant*	0	0	0	Inland	Africa, Asia, Australia	https://www.gbif.org/species/2495659
Columbidae	European Turtle Dove	Streptopelia turtur	Vagrant*	0	0	0	Inland	Europa, Africa	https://www.gbif.org/species/2495708
Cuculidae	Common Cuckoo	Cuculus canorus	Vagrant*	0	0	0	Inland	Europa, Africa	https://www.gbif.org/species/9372661
Diomedeidae	Kerguelen Petrel	Aphrodroma brevirostris	40000	1	1	0	Marine	Southern Ocean	https://www.gbif.org/species/2481439
Diomedeidae	Pintado Petrel	Daption capense	Common	1	1	0	Marine	Southern Ocean	https://www.gbif.org/species/2481545
Diomedeidae	Southern Royal Albatross	Diomedea epomophora	Regular	1	1	0	Marine	Southern Ocean	https://www.gbif.org/species/5229299
Diomedeidae	Northern Royal Albatross	Diomedea epomophora sanfordi	Rare	0	1	0	Marine	New Zealand	https://www.gbif.org/species/5229301
Diomedeidae	Wandering Albatross	Diomedea exulans	7300	1	1	0	Marine	Southern Ocean	https://www.gbif.org/species/5229302
Diomedeidae	Southern Fulmar	Fulmarus glacialoides	Fairly common	1	1	0	Marine	Southern Ocean	https://www.gbif.org/species/2481432
Diomedeidae	Blue Petrel	Halobaena caerulea	300000	1	1	0	Marine	Southern Ocean	https://www.gbif.org/species/2481597
Diomedeidae	Southern Giant Petrel	Macronectes giganteus	5500	1	1	0	Marine	Southern Ocean	https://www.gbif.org/species/2481515
Diomedeidae	Northern Giant Petrel	Macronectes halli	1300	1	1	0	Marine	Southern Ocean	https://www.gbif.org/species/2481516
Diomedeidae	Dark-mantled Sooty Albat	Phoebetria fusca	4400	1	1	0	Marine	Southern Ocean	https://www.gbif.org/species/2481376
Diomedeidae	Light-mantled Sooty Albat	Phoebetria palpebrata	700	1	1	0	Marine	Southern Ocean	https://www.gbif.org/species/2481374
Diomedeidae	White-headed Petrel	Pterodroma lessonii	Fairly common*	1	1	0	Marine	Southern Ocean	https://www.gbif.org/species/2481480
Diomedeidae	Great-winged Petrel	Pterodroma macroptera	40000	0	1	0	Marine	Southern Ocean	https://www.gbif.org/species/2481481
Diomedeidae	Soft-plumaged Petrel	Pterodroma mollis	30000	1	1	0	Marine	Southern Ocean	https://www.gbif.org/species/2481457
Diomedeidae	Shy Albatross	Thalassarche cauta	Fairly common	1	1	0	Marine	Southern Ocean	https://www.gbif.org/species/2481401
Diomedeidae	Yellow-nosed Albatross	Thalassarche chlororhynchos	15000	1	1	0	Marine	Southern Ocean	https://www.gbif.org/species/2481399
Diomedeidae	Grey-headed Albatross	Thalassarche chrysostoma	21800	1	1	0	Marine	Southern Ocean	https://www.gbif.org/species/2481408
Diomedeidae	Black-browed Albatross	Thalassarche melanophris	Common*	1	1	0	Marine	Southern Ocean	https://www.gbif.org/species/2481410
Diomedeidae	Salvin's Albatross	Thalassarche salvini	Rare	0	1	0	Marine	Australia, New Zealand, Chile	https://www.gbif.org/species/4847291
Diomedeidae	Antarctic Petrel	Thalassoica antarctica	Vagrant*	1	1	0	Marine	Southern Ocean	https://www.gbif.org/species/2481513
Falconidae	Unidentified falcon	Falco sp.	Vagrant*	0	0				
Hirundinidae	Common House Martin	Delichon urbicum	Vagrant*	0	0	0	Inland	Africa, Europe, Asia	https://www.gbif.org/species/2489214
Hirundinidae	Barn Swallow	Hirundo rustica	Vagrant*	1	1	1	Inland	Cosmopolitan	https://www.gbif.org/species/9515886
Hirundinidae	Brown-throated Martin	Riparia paludicola	Vagrant*	0	0	0	Inland	Africa, Asia	https://www.gbif.org/species/5230750
Hydrobatidae	Black-bellied Storm Petrel	Fregetta tropica	10000	1	1	0	Marine	Southern Ocean	https://www.gbif.org/species/2481979
Hydrobatidae	Grey-backed Storm Petre	Garrodia nereis	2000	1	1	0	Marine	Southern Ocean	https://www.gbif.org/species/2481988
Hydrobatidae	Wilson's Storm Petrel	Oceanites oceanicus	Common	1	1	0	Marine	Cosmopolitan	https://www.gbif.org/species/5229419
Laniidae	Red-backed Shrike	Lanius collurio	Vagrant*	0	0	0	Inland	Africa, Europe, Asia	https://www.gbif.org/species/7745240
Laridae	Kelp Gull	Larus dominicanus	260	1	1	0	Coastal	S Hemisphere	https://www.gbif.org/species/2481173
Laridae	Lesser Black-backed Gull	Larus fuscus	Vagrant*	1	0	0	Coastal	Primarily northern hemisphere	https://www.gbif.org/species/2481174
Laridae	Franklin's Gull	Leucophaeus pipixcan	Vagrant*	1	1	0	Coastal	N and S America mostly	https://www.gbif.org/species/5846430
Laridae	Sub-Antarctic Skua	Stercorarius antarcticus lonnbergi	1700	0	0	0	Marine	Southern Ocean	https://www.gbif.org/species/2481624
Laridae	Parasitic Jaeger	Stercorarius parasiticus	Rare	1	1	0	Coastal	Cosmopolitan	https://www.gbif.org/species/2481621
Laridae	Arctic Tern	Sterna paradisaea	Rare*	1	1	0	Coastal	mostly N Hemisphere	https://www.gbif.org/species/5229230
Laridae	Kerguelen Tern	Sterna virgata	110	0	0	0	Coastal	Kerguelen	https://www.gbif.org/species/5229219
Laridae	Antarctic Tern	Sterna vittata	60	1	1	0	Marine	mostly Southern Ocean	https://www.gbif.org/species/9606968
Laridae	Sabine's Gull	Xema sabini	Rare	0	1	0	Coastal	mostly N Hemisphere	https://www.gbif.org/species/2481221

Family	Vernacular Name	Scientific Name	Marion Status	Argentina	Chile	Overlap O.trinervis	Habitat	Distribution (GBIF)	GBIF Link
Motacillidae	Yellow Wagtail	Motacilla flava	Vagrant*	0	0	0	Inland	Africa, Europe, Asia, Alaska	https://www.gbif.org/species/9441286
Muscicapidae	Unidentified flycatcher	Muscicapa (striata?)	Vagrant*	0	0	0	Inland	Africa, Europe, Asia	https://www.gbif.org/species/2492576
Muscicapidae	Mountain Wheatear	Oenanthe monticola	Vagrant*	0	0	0	Inland	Africa	https://www.gbif.org/species/5231248
Passeridae	House Sparrow	Passer domesticus	Ship-assisted*	1	1	1	Inland	Cosmopolitan	https://www.gbif.org/species/5231190
Phalacrocoracidae	Crozet Shag	Phalacrocorax melanogenis	640	0	0	0	Coastal	Crozet and PEI endemic	https://www.gbif.org/species/6095258
Procellariidae	Great Shearwater	Ardenna gravis	Rare	1	1	0	Marine	Atlantic Ocean mostly	https://www.gbif.org/species/8596590
Procellariidae	Sooty Shearwater	Ardenna grisea	Fairly common	1	1	0	Marine	Cosmopolitan	https://www.gbif.org/species/8249990
Procellariidae	Cory's Shearwater	Calonectris diomedea	Rare	1	0	0	Marine	Atlantic	https://www.gbif.org/species/2481521
Procellariidae	Slender-billed Prion	Pachyptila belcheri	Fairly common	1	1	0	Marine	Southern Ocean	https://www.gbif.org/species/5229320
Procellariidae	Antarctic Prion	Pachyptila desolata	Fairly common	1	1	0	Marine	Southern Ocean	https://www.gbif.org/species/5229327
Procellariidae	Salvin's Prion	Pachyptila salvini	700000	0	1	0	Marine	Southern Ocean	https://www.gbif.org/species/5229328
Procellariidae	Fairy Prion	Pachyptila turtur	4000	1	1	0	Marine	Southern Ocean	https://www.gbif.org/species/5229332
Procellariidae	South Georgian Diving Pe	Pelecanoides georgicus	11000	1	0	0	Marine	Southern Ocean	https://www.gbif.org/species/2481429
Procellariidae	Common Diving Petrel	Pelecanoides urinatrix	20000	1	1	0	Marine	Southern Ocean	https://www.gbif.org/species/2481418
Procellariidae	White-chinned Petrel	Procellaria aequinoctialis	30000	1	1	0	Marine	mostly Southern Ocean	https://www.gbif.org/species/2481495
Procellariidae	Grey Petrel	Procellaria cinerea	12000	1	1	0	Marine	Southern Ocean	https://www.gbif.org/species/2481500
Procellariidae	Little Shearwater	Puffinus assimilis	Fairly common	1	1	0	Marine	Southern Ocean	https://www.gbif.org/species/5739275
Rallidae	Corncrake	Crex crex	Vagrant*	0	0	0	Inland	Europa, Africa	https://www.gbif.org/species/4408498
Scolopacidae	Common Sandpiper	Actitis hypoleucos	Vagrant*	0	0	0	Inland	Africa, Europe, Asia, Australia	https://www.gbif.org/species/2481800
Scolopacidae	Ruddy Turnstone	Arenaria interpres	Vagrant*	1	1	0	Coastal	Cosmopolitan	https://www.gbif.org/species/2481776
Scolopacidae	Curlew Sandpiper	Calidris ferruginea	Vagrant*	0	1	0	Inland	South Africa, Europe, Australia	https://www.gbif.org/species/2481741
Scolopacidae	Pectoral Sandpiper	Calidris melanotos	Vagrant*	1	1	1	Inland	mostly N and S America, Euro	https://www.gbif.org/species/2481744
Scolopacidae	Little Stint	Calidris minuta	Vagrant*	1	0	0	Inland	South Africa	https://www.gbif.org/species/2481749
Scolopacidae	Whimbrel	Numenius phaeopus	Vagrant*	1	1	0	Coastal	Cosmopolitan	https://www.gbif.org/species/2481784
Scolopacidae	Red Phalarope	Phalaropus fulicarius	Rare	1	1	0	Marine	N Hemisphere breeder, S Hen	https://www.gbif.org/species/5229390
Scolopacidae	Wood Sandpiper	Tringa glareola	Vagrant*	0	0	0	Inland	Africa, Europe, Asia, Australia	https://www.gbif.org/species/2481713
Scolopacidae	Common Greenshank	Tringa nebularia	Vagrant*	0	0	0	Inland	Africa, Europe, Asia, Australia	https://www.gbif.org/species/2481726
Scolopacidae	Terek Sandpiper	Xenus cinereus	Vagrant*	1	0	0	Coastal	Africa, Europe, Asia, Australia	https://www.gbif.org/species/2481703
Spheniscidae	King Penguin	Aptenodytes patagonicus	446000	1	1	0	Coastal	Southern Ocean	https://www.gbif.org/species/2481660
Spheniscidae	Southern Rockhopper Pe	Eudyptes chrysocome filholi	224000	0	0	0	Coastal	Southern Ocean	https://www.gbif.org/species/6178307
Spheniscidae	Macaroni Penguin	Eudyptes chrysolophus	750000	1	1	0	Coastal	Southern Ocean	https://www.gbif.org/species/2481643
Spheniscidae	Chinstrap Penguin	Pygoscelis antarcticus	Vagrant*	1	1	0	Coastal	Southern Ocean	https://www.gbif.org/species/2481664
Spheniscidae	Gentoo Penguin	Pygoscelis papua	3000	1	1	0	Coastal	Southern Ocean	https://www.gbif.org/species/2481666
Sulidae	Australasian Gannel	Morus serrator	Vagrant*	0	0	0	Coastal	Australia, New Zealand	https://www.gbif.org/species/5229212
Sylviidae	Willow Warbler	Phylloscopus trochilus	Vagrant*	0	0	0	Inland	Africa, Europe, Asia	https://www.gbif.org/species/2493052
Sylviidae	Common Whitethroat	Sylvia communis	Vagrant*	0	0	0	Inland	Africa, Europe, Asia	https://www.gbif.org/species/2492943

Supplementary Table S2. Sources of DNA material used from Aagesen, et al.[3], Burge, et al.[4], Kellermann and Udovicic[5], and Richardson, et al.[6]. Genbank numbers are listed for the *trn*L intron and the *trn*L-F spacer. Where the same number is listed for both DNA regions, this indicates that the full *trn*L-F region is deposited in GenBank. New sequences are indicated in bold and their voucher specimens listed.

**Taxon**	***trn*L intron (cd)**	***trn*L-F spacer (ef)**
Noltea africana	AJ390357	AJ390357
Adolphia infesta	AY460408	AY642142
Alphitonia excelsa	HQ325600	HQ325600
Colletia hystrix	AY409409	**MH263726**
	Argentina: Neuquén, *D.Medan 774* (BAA)	
Colletia paradoxa	AY460410	AY642143
Colletia ulicina	AJ390364	AJ390364
Discaria americana	AY460413	AY642144
Discaria articulata	AY460414	AY642145
Discaria chacaye	AY460415	AY642146
Discaria nitida	AY460418	AY642148
Discaria pubescens	AY460419	AY642149
Discaria toumatou	AY460420	AY642149
Ochetophila nana	AY460416	AY642147
Ochetophila trinervis	AY460421	AY642150
sp. Marion Island	**MH263730**	**MH263727**
	Sub-Antarctica: Marion Island, Western side of Black Lava, *W.A.Haddad 1024* (PRU 121568)	
Kentrothamnus weddellianus	AY460422	AY642152
Retanilla ephedra	AY460423	**MH263728**
	Argentina: Buenos Aires, *D.Medan s.n.* (BAA)	
Retanilla patagonica	AY460424	AY642153
Retanilla stricta	AY460425	**MH263729**
	Chile: Colchagua, *D.Medan 790* (BAA)	
Retanilla trinervia	AY460426	AY642154
Schistocarpaea johnsonii	AJ390349	AJ390349
Spyridium parvifolium	EF528526	EF528526
Trevoa quinquenervia	AY460427	AY642155
